# Fatal Leishmaniasis in the Absence of TNF Despite a Strong Th1 Response

**DOI:** 10.3389/fmicb.2015.01520

**Published:** 2016-01-22

**Authors:** Phillip D. Fromm, Jessica C. Kling, Annika Remke, Christian Bogdan, Heinrich Körner

**Affiliations:** ^1^Comparative Genomics Centre, James Cook University, TownsvilleQN, Australia; ^2^Menzies Institute for Medical Research Tasmania, HobartTAS, Australia; ^3^Blumenthal Group, The University of Queensland Diamantina Institute, Translational Research Institute, WoolloongabbaQLD, Australia; ^4^Mikrobiologisches Institut – Klinische Mikrobiologie, Immunologie und Hygiene, Friederich-Alexander-Universität Erlangen-Nürnberg, Universitätsklinikum ErlangenErlangen, Germany

**Keywords:** cutaneous leishmaniasis, IFN-γ, tumor necrosis factor, T cell subtypes, mouse models

## Abstract

Induction of inducible nitric oxide synthase in mononuclear phagocytes by IFN-γ and innate tumor necrosis factor (TNF) provide the basis for an effective immune response to the intracellular parasite *Leishmania (L.) major*. In previous experiments, we observed a fatal visceral form of leishmaniasis in *L. major*-infected C57BL/6 TNF^-/-^ mice. To further delineate the protective function of TNF and its receptor requirements, we comparatively assessed *L. major*-infected C57BL/6 mice that were either deficient for membrane *and* soluble TNF (*Tnf*^-^*^/^*^-^), for soluble TNF alone (*memTnf^Δ/Δ^*), or the TNF receptors type 1 (*Tnfr1*^-^*^/^*^-^) or type 2 (*Tnfr2*^-^*^/^*^-^). We detected locally and systemically increased levels of the cytokine IFN-γ in the absence of the TNF-TNFR1-signaling pathway. An analysis of transcription factors and cytokines revealed that activated *Tnf*^-^*^/^*^-^ CD4^+^ T cells displayed a highly active Th1 phenotype with a strong usage of the T cell receptor Vβ5.1/2. From these data we conclude that the fatal outcome of *L. major* infection in *Tnf*^-^*^/^*^-^ mice does not result from a skewed or deficient Th1 differentiation.

## Introduction

Cutaneous leishmaniasis is caused by different species of the protozoan parasite genus *Leishmania* (L.) such as the “old world” species of *Leishmania major* (Reithinger et al., 2007; Schonian et al., 2008), which are transferred by the bite of a sand fly (Sacks and Perkins, 1985). In the case of a *L. major* infection, the clinical manifestation is limited to a local skin lesion that heals without further treatment. However, depending on the host immune response and the parasite species and strain, chronic, non-healing skin ulcers and widespread tissue destruction or even systemic parasite dissemination have also been observed (Reithinger et al., 2007).

The immune response to *L. major* in the skin and draining LNs has been analyzed extensively using high- or low-dose mouse infection models (Sacks and Noben-Trauth, 2002; Bogdan, 2008; Liese et al., 2008), but is still only partly understood. In genetically self-healing C57BL/6 mice a protective immune response against *L. major* occurs that is characterized by an initial boost of interferon (IFN)-α/β (Diefenbach et al., 1998), the rapid induction of IL-12 (Scharton-Kersten et al., 1995), the production of IFN-γ by NK cells and the differentiation of Th1 cells (Liese et al., 2007; Lykens et al., 2010; Prajeeth et al., 2011). IFN-γ drives macrophages to upregulate the enzyme inducible nitric oxide synthase (iNOS, NOS2; Ding et al., 1998) to produce large amounts of the leishmanicidal effector molecule nitric oxide (NO) from L-arginine (Deng et al., 1993), a process synergistically supported by tumor necrosis factor (TNF; Ding et al., 1998). Synthesis of NO is essential for the resolution of *L. major* infections *in vivo* (Liew et al., 1990; Stenger et al., 1994, 1996; Diefenbach et al., 1998; Prajeeth et al., 2011). This well established chain of immunological events in the resistant C57BL/6 mouse contrasts with the immune response to *L. major* in the genetically susceptible (i.e., non-healing) BALB/c mouse which shows an early and sustained IL-4 expression that results in a progressive infection (Sacks and Noben-Trauth, 2002). The observed dichotomy in the cytokine response led to the development of the Th1–Th2 model of T cell differentiation, which interprets genetic differences of cytokine expression as causal for the disparate clinical outcomes observed in experimental cutaneous Leishmaniasis (Mosmann and Coffman, 1989). However, more recent work has demonstrated that this classical model is too simplistic, as newly characterized T cell populations (e.g., Th17 cells, regulatory T cells) (Alexander and Brombacher, 2012), early chemokine expression (Roebrock et al., 2014) and differential wound healing mechanisms (Baldwin et al., 2007) need to be taken into account when discussing genetic and immunological reasons for susceptibility to *L. major*. In addition, in TNF-deficient mice *L. major*-specific T cells displayed strong *in vitro* IFN-γ expression, but *in vivo* failed to achieve control of *L. major* as TNF^-/-^ mice succumbed to the infection within 6–7 weeks (Wilhelm et al., 2001). These and other results, which contradicted the classical Th1/Th2 dichotomy (Belosevic et al., 1989; Anderson et al., 2005), suggested that the presence of IFN-γ is necessary, but not sufficient for the control of *L. major in vivo*.

To further address the role of TNF for the innate and adaptive response to *L. major* and to reconcile previous discrepant results obtained with TNF- or TNF-receptor-deficient mouse strains of different genetic origins (Vieira et al., 1996; Nashleanas et al., 1998; Chakour et al., 2003), we infected mice deficient for membrane *and* soluble TNF (*Tnf*^-^*^/^*^-^), for soluble TNF alone (*memTnf^Δ/Δ^*), for TNF receptor type 1 (*Tnfr1*^-^*^/^*^-^), or type 2 (*Tnfr2*^-^*^/^*^-^) on a pure C57BL/6 background to comprehensively assess the course of leishmaniasis. Subsequently, we compared the expression of IFN-γ and the underlying T cell response in Wt and *Tnf*^-^*^/^*^-^ mice to analyze and quantify the adaptive immune response in the absence of TNF in more detail.

## Materials and Methods

### Mice

The gene-targeted C57BL/6 mouse strains deficient for both soluble and membrane TNF (*Tnf*^-^*^/^*^-^) or for soluble TNF only (*memTnf^Δ/Δ^*) were generated on a genetically pure C57BL/6 (Wt) background (Körner et al., 1997; Ruuls et al., 2001). The *Tnfr1*^-/-^ (Jackson stock number: 003242) and *Tnfr2*^-^*^/^*^-^ mice (Jackson stock number: 002620) were obtained from Jackson Laboratories and had been backcrossed more than 10 times or had been established on a C57BL/6 background, respectively (Peschon et al., 1998). The screening procedure followed the protocols published previously (Körner et al., 1997; Peschon et al., 1998). All animals were kept under specific pathogen-free conditions at the Animal Research Facilities of the University of Tasmania, Australia, the Comparative Genomics Centre, James Cook University, Australia, and the Institute for Clinical Microbiology, Immunology and Hygiene at the University Hospital of Erlangen, Germany. All experiments followed protocols approved by the animal ethics committees of James Cook University, Townsville, the University of Tasmania, Hobart, Tasmania and the Government of Mittelfranken, Germany. Mice of an age of 8–12 weeks were used in all experiments.

### Parasites and Infection

The virulent *L. major* isolate MHOM/IL/81/FE/BNI (Solbach et al., 1986; Stenger et al., 1996) was maintained through serial passage in BALB/c mice *in vivo* and cultured *in vitro* in Novy-Nicolle-MacNeal blood agar slants in RPMI1640 medium supplemented with 10% new born calf serum, penicillin/streptomycin, non-essential amino acids and 10 mM HEPES, all supplied by Invitrogen Life Technologies (Mount Waverly, Australia). For infection, stationary phase *L. major* promastigotes were used between passage 2 and 6 and 3 × 10^6^ parasites were injected in a volume of 40 μl into one hind footpad. The infection site was monitored daily and the increase in lesion size was determined weekly by measuring the footpad thickness with a metric caliper (Kroeplin Schnelltaster, Schluechtern, Germany). The percentage of increase in footpad thickness was determined by the formula (thickness of infected footpad minus thickness of non-infected footpad/mean thickness of non-infected footpad) × 100. The parasite burden (per gram of tissue) was determined at day 28 post infection (p. i.) using a limiting dilution method and L-Calc software version 1.1 (Stem Cell Technologies, www.Stemcell.com) which performs a generalized Pearson Chi-squared test (Wilhelm et al., 2001).

### Flow Cytometry

Draining popliteal (p) lymph nodes (LN) or footpad lesions were incubated with collagenase D (1 mg/ml, Roche Products Australia, Brisbane, QLD, Australia) and DNAse 1 (100 U/ml, Sigma–Aldrich) for 30 min at 37°C and disrupted between frosted glass slides yielding single cell suspensions. Cells were filtered through 60 μm nylon meshes or 40 μm cell strainers (BD Biosciences, Sydney, NSW, Australia) to remove tissue debris. Prior to FACS staining the cells were blocked with either anti-CD16/32 antibody (clone 2.4G2, eBioscience, San Diego, CA, USA) or 10% rat serum (IMVS, Adelaide, SA, Australia). Cells were stained with rat-anti-mouse antibodies specific for B220 (RA3-6B2, Pacific Blue, or APC-Cy7), CD4 (RM4-5, PerCP-Cy5.5, or Pacific Blue), CD8 (53–6.7, Pacific Blue), CD25 (PC61, APC; 7D4, FITC), CD44 (IM7, PeCy7), CD62L (MEL-14, APC), GITR (DTA-1, PeCY7), Vß4 TCR (KT4, PE), Vß5.1/5.2 TCR (MR9-4, FITC) CD3𝜀 (145-2C11, PE-Cy7), IL-17 (TC11-18H10, PE), IL-4 (11B11, PE), IFN-γ (XMG1.2, Alexa Fluor-488) and with Armenian hamster-anti-mouse antibodies specific for TCR β-chain (H57-597, APC or biotin/Streptavidin Pacific Orange). Multicolour staining of single cells for surface antigens was performed as published (Wilhelm et al., 2001). Data were acquired using a Cyan ADP (Beckman Coulter, Fullerton, CA, USA). Analyses were performed using FlowJo^®^ version 8.86 (Tree Star Inc.).

### Cell Isolation

CD4^+^ T cells were isolated from draining LN using antibody labeled magnetic beads (Miltenyi Biotec Australia, Sydney, NSW, Australia). Briefly, the LN were disrupted between frosted glass slides yielding single cell suspensions. The cells were washed with PBS/0.1% BSA and incubated with anti-CD4 beads (Miltenyi Biotec) for 15 min on ice. After labeling, all CD4^+^ cells (naïve as well as activated) were isolated using a standard lymphocyte protocol on a fully automated autoMacs Pro Separator (Miltenyi Biotec) which was provided by the manufacturer.

### Cytokine Analysis

Intracellular cytokine staining was performed on antigen-stimulated T cells. Cells that included CD4^+^ T cells and antigen-presenting cells were re-stimulated in 96 well plates (Sarstedt Australia, Mawson Lakes, SA, Australia) at a density of 5 × 10^5^ cells using freeze-thawed *L. major* antigen (MOI equivalent = three) for 72 h in RPMI1640 medium supplemented with 10% new born calf serum, penicillin/streptomycin, non-essential amino acids, and 10 mM HEPES, all supplied by Invitrogen Life Technologies (Mount Waverly, Australia). For the last 6 h of culture the cells were kept in the presence of PMA (20 ng/ml)/ionomycin (1 μg/ml) and Golgi-Stop^TM^ (BD Biosciences, 4 μg/ml). Subsequently, the cells were stained for surface antigens, fixed, permeabilised using FoxP3-FixPerm buffer (Biolegend, Perth, Australia) and stained for IFN-γ, IL-4, and IL-17 following the manufacturers’ instructions.

Serum was collected from experimental animals weekly during the course of infection. Cytokine levels were measured in mouse serum and tissue culture supernatants using mouse inflammation cytokine bead array (CBA, BD Biosciences). CBA were acquired on the ARIA II and FCS 2.0 files were analyzed using FCAP Array (Soft Flow Inc, Burnsville, MN, USA).

Real-time RT-PCR was performed with total RNA extracted from footpad lesions using Trizol (Invitrogen). Following DNase treatment of RNA (using RQ1 RNase free DNase; Promega, Sydney Australia) cDNA synthesis was performed with Superscript III reverse transcriptase (Invitrogen). Gene expression analysis was performed using SYBR-green-ER qPCR kit (Invitrogen) or Brilliant II SYBR-green (Agilent Technologies, Integrated Sciences, Willoughby, NSW, Australia), run on a Corbett Rotor Gene 6000 (Qiagen, Doncaster, Australia) and analyzed using REST 2009 gene expression software (Qiagen) to determine relative expression of genes. PCR primers were designed using Vector NTI (Invitrogen): *β-actin-*forward: AAT CCT GTG GCA TCC ATG AAA C, β*-actin*-reverse: CGC AGC TCA GTA ACA GTC CG; *GAPDH-* forward: GTG AAG GTC GGT GTG AAC GG, *GAPDH-*reverse: ATG TTA GTG GGG TCT CGC TCC; *Gata3*-forward: GAG GTG GAC GTA CTT TTT AAC AT, *Gata3*-reverse: GGC ATA CCT GGC TCC CGT; *Hprt*-forward: GTT GGT TAC AGG CCA GAC TTT GTT G, H*prt*-reverse: GAG GGT AGG CTG GCC TAT AGG CT; *Il-10*-forward: GGT TGC CAA GCC TTA TCG GA, *Il-10*-reverse: ACC TGC TCC ACT GCC TTG CT; *Il-17A*-forward: TCT GTG TCT CTG ATG CTG TTT GC, *Il17A*-reverse: ACG GTT GAG GTA GTC TGA GGG C; *Ifn-γ*-forward: AGA GCC AGA TTA TCT CTT TCT AC, *Ifn-γ*-reverse: CTT TTT TCG CCT TGC TGC TG; *Rorγt*-forward: CCG CTG AGA GGG CTT CAC, *Rorγt*-reverse: TGC AGG AGT AGG CCA CAT TAC A; *Tbx21*-forward: CAA CAA CCC CTT TGC CAA AG, *Tbx21*-reverse: TCC CCAA GCA AGT TGA CAGT.

### Statistics

Statistical analysis was performed using a non-parametric Mann Whitney *U*-test, a Kruskall–Wallis with Dunn’s multiple comparison test or a two-way ANOVA test with Bonferroni correction to test for multiple hypotheses. Mean values (±SEM) are shown. Analysis was performed using GraphPad Prism 5.0 for MacIntosh (GraphPad Software, San Diego, CA, USA, www.graphpad.com). Each experimental group was compared to Wt controls. Statistical *p* values of *p* < 0.05 were considered to be significant (with ^∗^*p* < 0.05 and ^∗∗^*p* < 0.01, respectively).

## Results

### Clinical Course of *L. major* Infection in the Absence of TNF or its Receptors

**FIGURE 1:**
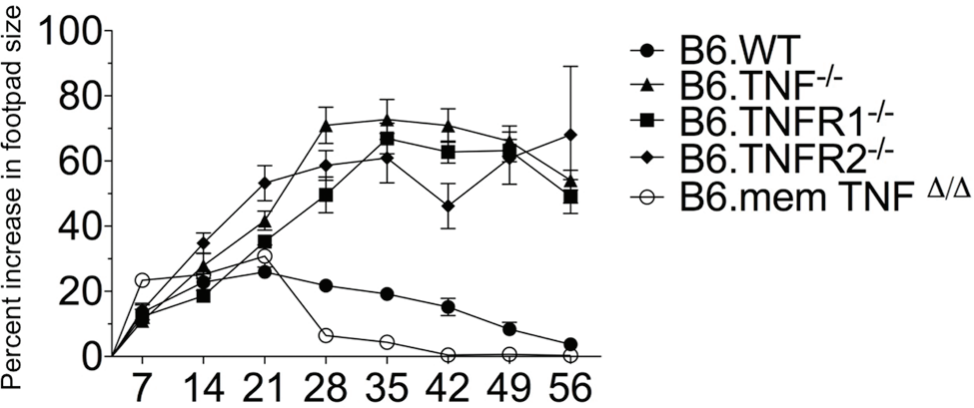
**Course of infection in *Tnf-* and *Tnfr*-negative mouse strains.** The lesion sizes of *Tnf*^-^*^/^*^-^ (*n* = 34), *memTnf^Δ/Δ^* (*n* = 5), *Tnfr1*^-^*^/^*^-^ (*n* = 27), and *Tnfr2*^-^*^/^*^-^ (*n* = 27) mice, which had been infected subcutaneously with *Leishmania major* in one hind footpad were determined and compared to Wt (*n* = 36). The data are presented as percent increase in footpad lesion size. The combined results of four experiments are shown as mean (±SEM) and the number of animals stated above is the maximal number at the beginning of the experiment. The differences in clinical outcome were combined for analysis using a two-way ANOVA with Bonferroni correction to test for multiple hypotheses.

The published clinical outcomes of *L. major* infection in *Tnfr1*^-^*^/^*^-^ and *Tnfr2*^-^*^/^*^-^ mice (Vieira et al., 1996; Nashleanas et al., 1998) were significantly different from the infection of *Tnf*^-^*^/^*^-^ mice (Wilhelm et al., 2001). It has been shown earlier using bone marrow reconstitution that changes in the lymphoid organs due to the absence of TNF signaling only have a minimal influence on the clinical outcome of the infection (Wilhelm et al., 2001). Therefore, this could reflect the use of different *L. major* strains (Ritter et al., 2004) or the genetic heterogeneity of the mouse strains. Therefore, we infected *Tnf*^-^*^/^*^-^, *Tnfr1*^-^*^/^*^-^, *Tnfr2*^-^*^/^*^-^, and *memTnf^Δ/Δ^* strains (Körner et al., 1997; Peschon et al., 1998; Ruuls et al., 2001) with the pathogenic *L. major* isolate BNI and monitored the course of disease (**Figure 1**). In accordance with previously described results obtained with a different strain of *L. major* (Allenbach et al., 2008), infected *memTNF^Δ/Δ^* mice, which express the membrane form but lack the ability to release soluble TNF, and Wt control mice showed a comparable course of infection (**Figure 1**) and were able to control the infection. In contrast, *L. major*-infected *Tnf*^-^*^/^*^-^, *Tnfr1*^-^*^/^*^-^, and *Tnfr2*^-^*^/^*^-^ mice all developed similar, large skin lesions strikingly different from the Wt control mice. At day 56 all control mice had resolved their lesions, whereas infected *Tnf-* and *Tnfr1*-deficient mice had to be euthanized in accordance with animal ethics considerations because the animals developed signs of systemic distress. *L. major*-infected *Tnfr2-*deficient mice developed large lesions, which were comparable in size to those in TNF or TNFR1 mice, but ultimately controlled the infection (**Figure 1** and data not shown). The wildtype controls [C57BL/6, shown as B6.WT (**Figure 1**) and BALB/c (data not shown)] exhibited the expected symptoms with B6.Wt controlling the infection whereas BALB/c succumbing to a progressive infection. From these data we conclude that TNF and TNFR1 are both essential for controlling an infection with *L. major*, whereas TNFR2 only plays a contributory role. Furthermore, membrane TNF alone is sufficient to convey protection.

### Induction of IFN-γ Response in *Tnf*^-/-^ Mice During *L. major* Infection

**FIGURE 2:**
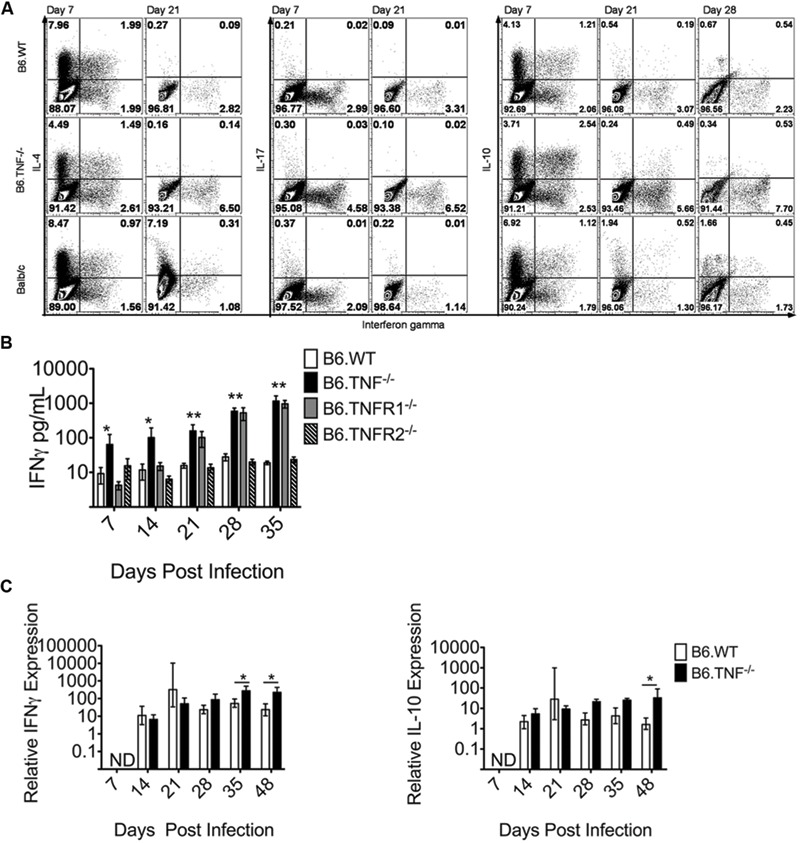
**TNF-TNFR1 signaling pathway deficiency results in elevated IFN-γ expression after *L. major* infection. (A)** Cytokine expression in CD4^+^ T cells from popliteal draining LNs of *L. major* infected Wt, *Tnf*^-^*^/^*^-^ and BALB/c mice was analyzed. The use of the *Tnf*^-^*^/^*^-^ mice constitutes the disruption of the complete TNF-TNFR1 signaling pathway. Intracellular flow cytometry was used to determine the proportional expression of IFN-γ, IL-4, IL-17, and IL-10 in CD4^+^ T cells at day 7 and 21 (and in the case of IL-10 also day 28) p. i. The experiment was performed two times independently and a representative staining is shown. **(B)** The concentration of IFN-γ in the serum of *L. major* infected Wt, *Tnf*^-^*^/^*^-^, *Tnfr1*^-^*^/^*^-^, and *Tnfr2*^-^*^/^*^-^ was determined. The data are presented as mean (±SEM; *n* = 5–6 per genotype). Results are representative of at least 3 independent experiments. **(C)** The relative expression of IFN-γ and IL-10 in the footpad lesion of infected Wt or *Tnf*^-^*^/^*^-^ mice was compared to uninfected controls. Relative expression was calculated relative to β-actin as described (Meissner et al., 2003). The data are presented as median (±SEM; *n* = 3–5 mice, representative of two independent experiments). The white bar represents Wt, the black bar *Tnf*^-^*^/^*^-^ mice. The non-parametric Mann–Whitney *U*-test was used for analysis of experiments displayed in **(B,C)** to test for statistical differences (^∗^*p* < 0.05 ^∗∗^*p* < 0.01).

The cytokine milieu is essential for induction of a leishmanicidal response in macrophages and for the establishment of protective immunity. To determine the impact of a TNF or TNF receptor deficiency on the development of the adaptive immune response, we first infected *Wt, Tnf*^-^*^/^*^-^ and BALB/c mice with *L. major* and analyzed CD4^+^ T cells from popliteal draining lymph nodes (pLN) for their expression of IL-4, IFN-γ, IL-17, and IL-10. We observed a relatively early, strong IL-4 expression in all mouse strains on day 7 p. i., which had largely disappeared on day 21 p. i. (**Figure 2A**). IFN-γ was present in all three genotypes with a significantly elevated expression in TNF^-/-^ (see **Figure 2B**), while another pro-inflammatory cytokine, IL-17, was hardly detectable in CD4^+^ T cells of any strain at the analyzed time-points (**Figure 2A**). The expression of IL-10 was determined after 7, 21, and 28 days and was very similar to IL-4 expression with a strong presence at day 7 p. i., and fast down-regulation thereafter (**Figure 2A**). Both IL-4 and IL-10 were co-expressed with IFN-γ in 1 – 2% of all cells (**Figure 2A**). This finding is likely to reflect a random heterogeneity during the differentiation of Th1 and Th2 cells at the beginning of the adaptive immune response (Morris et al., 1992), as the IL-4-, IL-10-, and IFN-γ-co-expressing cells were only detectable at day 7 p. i.

A quantification of the IFN-γ expression in the serum of all genotypes at days 7, 14, 21, 28, and 35 p. i. showed a significantly increased concentration of IFN-γ throughout the course of infection exclusively in both *Tnf*^-^*^/^*^-^ and *Tnfr1*^-^*^/^*^-^ mice (**Figure 2B**). The expression level of IFN-γ mRNA in the footpad lesions was initially comparable between infected *wt* and *Tnf*^-^*^/^*^-^ mice, but was significantly increased in *Tnf*^-^*^/^*^-^ mice after day 35 of infection (**Figure 2C**). IL-10 expression relative to the housekeeping gene was also increased significantly at day 35 p. i. (**Figure 2C**). However, as demonstrated by flow cytometry, the protein expression of IL-10 was substantially lower than of IFN-γ which points to an unexpectedly stable and even enhanced Th1-type response in the absence of TNF (**Figure 2C**).

### Expression Analysis of Transcription Factors and Cytokines in Activated CD4^+^ T cells in *L. major*-Infected Wt and *Tnf*^-/-^ Mice

**FIGURE 3:**
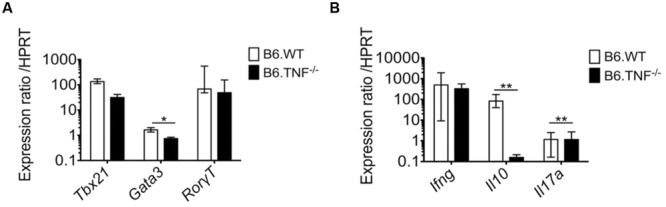
**Expression of *Tbx21* is unchanged while expression of *Gata3* is reduced in activated *Tnf*-deficient CD4^+^ T cells.** Naïve (CD62L^+^ CD44^-^) as well as activated (CD62L^-^ CD44^+^) CD4^+^ T cells from Wt or *Tnf*^-^*^/^*^-^ mice were isolated from the spleens of individual mice at day 50 after *L. major* infection. Quantitative real time RT-PCR was performed to analyze the relative mRNA expression of transcription factors **(A)** and cytokines **(B)**. The expression of the different genes was normalized with the house keeping gene hypoxanthine guanine phosphoribosyl transferase (HPRT). Data are presented as median (±SEM) of the mRNA expression ratio of activated vs. naive CD4^+^ T cells (*n* = 5–6 mice, one representative of two independent experiments is shown) (^∗^*p* < 0.05, ^∗∗^*p* < 0.01).

The polarization of T cell responses is strongly regulated by the balance of a number of transcription factors such as *Tbx21* (*Tbet*) and *Gata3* that regulate the expression of IFN-γ and IL-4, respectively (Zheng and Flavell, 1997; Szabo et al., 2000, 2002). To investigate a potential cause for the increased IFN-γ expression in the absence of the TNF-TNFR1 signaling pathway during the course of *L. major* infection, we isolated populations of both activated (CD62L^-^ CD44^+^) and naïve CD4^+^ T cells (CD62L^+^ CD44^-^) from the spleens of *Wt* and *Tnf*^-^*^/^*^-^ mice at day 50 p. i. and analyzed the expression level of several genes. The relative increase in *Tbx21* expression in activated T cells of both genotypes (*Wt*: mean = 134 [range 111.43–170.87]; *Tnf*^-^*^/^*^-^*:* mean = 34 [range 23.7–41.56]) was in agreement with the observed increase in *Ifn-γ* expression (497-fold upregulation in *wt* mice and 324-fold upregulation in *Tnf*^-^*^/^*^-^ mice) (**Figure 3B**). In contrast, the expression of *Gata3*, which directs both IL-4 and IL-10 production (Zheng and Flavell, 1997; Shoemaker et al., 2006), was only weakly induced (1.6-fold) in activated wt CD4^+^ T cells and decreased (1.42-fold) in activated *Tnf*^-^*^/^*^-^ CD4^+^ T cells as compared to the respective naive CD4^+^ T-cell populations (**Figure 3A**). This was associated with a dramatic reduction of the IL-10 mRNA expression (**Figure 3B**). Additionally, we analyzed the expression of the transcription factor *Rorγt (Rorc)* which is characteristic for the pro-inflammatory Th17 T cell subset (Yang et al., 2008). At this late stage of infection it was up-regulated in activated CD4^+^ T cells of both *wt* (mean = 67.9-fold) and *Tnf*^-^*^/^*^-^ (mean = 48.6-fold) mice to a similar extent (**Figure 3A**), although expression of IL-17A was generally low and barely detectable either by real-time PCR (**Figure 3B**) or by intracellular cytokine staining (**Figure 2A**).

Thus, only the *Gata3* gene was expressed differently between the genotypes and consequently IL-10 expression was dramatically reduced in infected *Tnf*^-^*^/^*^-^ mice. These data further corroborate that mice lacking the TNF-TNFR1 signaling pathway show an exaggerated Th1 response but nevertheless are unable to resolve the infection.

### Analysis of CD4^+^ T cell Activation During Leishmaniasis

**FIGURE 4:**
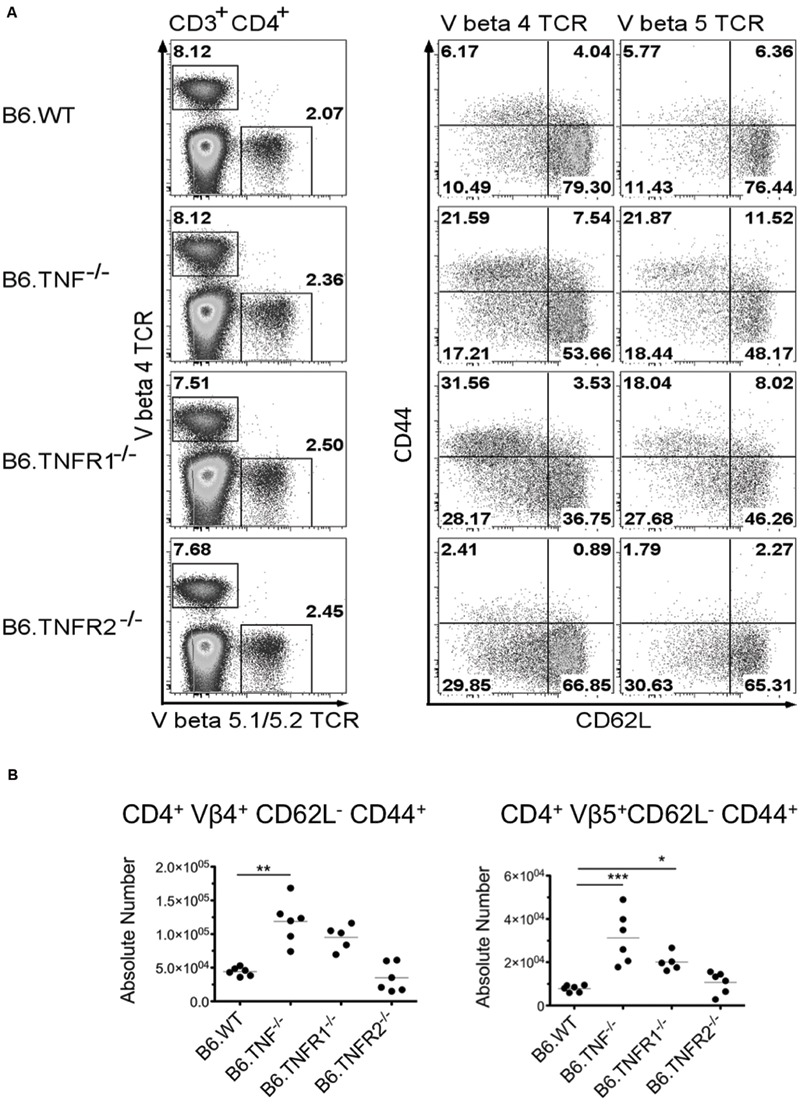
**Expansion and activation of Vβ4 and Vβ5.1/5.2 TCR repertoire occurs in the absence of TNF or TNFR1 but not TNFR2.** Wt mice or mice lacking TNF, TNFR1, or TNFR2 were infected with 3 × 10^6^ *L. major* promastigotes and draining pLN were harvested at day 21 p. i. **(A)** *Tnf*- and *Tnfr1*-deficient mice showed increased activation of CD4^+^ T cells expressing either Vβ4 or Vβ5.1/5.2 TCR chains. One of two representative experiments is shown. **(B)** Increased activation was correlated with increased absolute numbers of both Vβ4 or 5 restricted activated (CD62L^-^ CD44^+^) CD4^+^ T cells in the draining pLN by a comparable size of the organs. Statistical comparisons were performed using a Kruskal–Wallis Test with Dunns correction; *n* = 5–6 per genotyope ^∗^*p* ≤ 0.05, ^∗∗^*p* ≤ 0.01, ^∗∗∗^*p* < 0.001.

Both susceptible and resistant strains of mice were reported to develop strong antigen-specific responses to epitopes of the Leishmania homolog of receptors for activated C-kinase antigen (LACK) with a predominant stimulation of Vα8 Vβ4-positive T cells, whereas the Vβ5-expressing T cell population did not expand (Launois et al., 2007). Therefore, we followed the expansion of Vβ4 TCR^+^ CD4^+^ T cells and concurrently analyzed the Vβ5.1/5.2 TCR^+^ CD4^+^ T cells (Launois et al., 1997) in the draining pLNs of *Wt, Tnf-, Tnfr1-*, or *Tnfr2*-deficient mice. The activation of these T cells was studied using two classical activation markers of peripheral CD4^+^ T cells, CD62L and CD44 (Tough et al., 1996). Within the draining LN isolated from *Wt, Tnf*^-^*^/^*^-^, *Tnfr1*^-^*^/^*^-^, and *Tnfr2*^-^*^/^*^-^ mice, a comparable proportion of T cells was Vβ4 TCR^+^ CD4^+^ (**Figure 4A**). However, the population of Vβ4 TCR^+^ CD4^+^ T cells from *Tnf*^-^*^/^*^-^ and *Tnfr1*^-^*^/^*^-^ mice contained a significantly larger proportion of activated (CD62L^-^ CD44^+^) T cells (**Figure 4A**) and showed also an increase in the absolute numbers of activated Vβ4 TCR^+^ CD4^+^ T cells within the draining pLN (**Figure 4B**). Interestingly, Vβ5.1/5.2 TCR^+^ CD4^+^ T cells from *Tnf*^-^*^/^*^-^ and *Tnfr1*^-^*^/^*^-^ mice showed a similar increase in activation as shown for Vβ4 TCR^+^ CD4^+^ T cells, pointing to a strong non-specific activation of the T cell compartment in these genotypes (**Figure 4B**). In Wt as well as *Tnfr2*-deficient mice neither Vβ4 TCR^+^ T cells nor Vβ5 TCR^+^ T cells displayed significant activation at this time-point of analysis. By day 50 p. i. the T cell compartment of *Tnf*^-^*^/^*^-^ mice had collapsed and both Vβ4 and Vβ5.1/5.2 TCR^+^ CD4^+^ T cell subsets were reduced within the pLN (data not shown).

## Discussion

The TNF signaling pathways, especially the TNF-TNFR1 pathway, are essential in generating an inflammatory response and mediating resistance to infection by intracellular pathogens such as *L. major* (Wilhelm et al., 2001; Körner et al., 2010). In the absence of the pro-inflammatory cytokine TNF the protection against *L. major* is severely impaired resulting in a progressive infection and eventually a fatal outcome. Interestingly, using reciprocal bone marrow chimeras we could show that the ability of hemopoietic cells to express TNF confers protection (Wilhelm et al., 2001). The relatively subtle structural changes of lymphoid organs in *Tnf-* and *Tnfr1-*gene deficient mice were not relevant with regard to protection against *L. major* (Wilhelm et al., 2001).

In the present study we have analyzed the clinical course of *L. major* infection in C57BL/6 mouse strains deficient for TNF, soluble TNF (*memTnf^Δ/Δ^*), TNFR1 or TNFR2 and could show that the outcome was fatal in the absence of TNF and TNFR1. A deficiency of soluble TNF did not impair the healing phenotype associated with the C57BL/6 background as long as the membrane-bound cytokine was still expressed. This is in agreement with earlier studies that showed that transmembrane TNF expressed on CD4^+^ T cells was sufficient to induce a protective anti-Leishmania immune response (Birkland et al., 1992; Allenbach et al., 2008). A *Tnfr2*-deficiency resulted in the development of large skin lesions, which, however, ultimately was resolved. The fatal outcome observed in *L. major* infected *Tnf-* or *Tnfr1*-deficient mice occurred despite a seemingly intact adaptive immune response and an over-expression of IFN-γ.

Our results describing a fatal course of *L. major* infection in TNF^-/-^ (Wilhelm et al., 2001) contrast with two other studies published in experimental cutaneous leishmaniasis, which either used anti-TNF antibodies in C3H/HeN mice (Titus et al., 1989) or a *Tnf*^-^*^/^*^-^ strain on a different mouse background (Chakour et al., 2003). In both cases an aggravated clincial course of infection was observed, but the mice resolved the lesions and survived. Similarly, our findings on the contribution of TNFR1 or TNFR2 to the protective response to *L. major* also differ from several earlier reports. Vieira et al. (1996) used a *Tnfr1*^-^*^/^*^-^ strain generated on a 129Sv background and showed that these mice survived the infection and eliminated the parasites but did not completely resolve the skin swelling and pathology. Infection of *Tnfr2*^-^*^/^*^-^ mice on a mixed 129Sv × C57BL/6 background showed that this receptor was dispensable for the control of *L. major* (Nashleanas et al., 1998) and, finally, an infection of a *Tnfr1/2* double-deficient 129Sv × C57BL/6 mouse followed the *Tnfr1*^-^*^/^*^-^ phenotype (Nashleanas et al., 1998). In the present study, we compared *memTnf^Δ/Δ^, Tnf*^-^*^/^*^-^, *Tnfr1*^-^*^/^*^-^, and *Tnfr2*^-^*^/^*^-^ mice on an identical C57BL/6 genetic background using the *L. major* parasite strain BNI to account for differences caused by the genetic variability of the infected mouse strain and the parasite strain used (Ritter et al., 2004). In our experiments the previously observed phenotypes could not be replicated with the exception of the resistance of *memTnf^Δ/Δ^* to the infection. Both *Tnfr1*^-^*^/^*^-^ and *Tnfr2*^-^*^/^*^-^ mice displayed a lesion development comparable to *Tnf*^-^*^/^*^-^ mice suggesting overlapping roles for the two receptors in the pathogenesis of leishmaniasis. Interestingly, *Tnfr2*^-^*^/^*^-^ mice ultimately survived, whereas both *Tnf*^-^*^/^*^-^ and *Tnfr1*^-^*^/^*^-^ mice developed signs of severe systemic disease and succumbed to the infection.

Experimental cutaneous leishmaniasis in genetically inbred mice was the first model which showed a mouse strain-dependent polarization of IL-4 and IFN-γ production by CD4^+^ T cells correlating with either disease susceptibility or resistance (Heinzel et al., 1989) and therefore formed the basis of the Th1 and Th2 paradigm (Mosmann and Coffman, 1989). In our experiments, the expression of IL-10 paralleled the expression of IL-4. Additionally, we detected a small percentage of both IFN-γ/IL-10 and IFN-γ/IL-4 double positive T cells at day 7 p. i. which disappeared soon after. These cells could be part of a regulatory subpopulation or represent random heterogeneity during T cell differentiation at the beginning of the antigen-specific immune response (Morris et al., 1992). The major argument for a central role of IFN-γ in resistance to *L. major* infection has been its polarizing influence on CD4^+^ T cell differentiation (Heinzel et al., 1989; Laouar et al., 2005) and its activating effect on macrophages with the induction of leishmanicidal NO *in vitro* (Liew et al., 1990) and *in vivo* (Stenger et al., 1994, 1996; Diefenbach et al., 1998). This has been tested by administration of anti-IFN-γ mAbs to C3H/HeN mice which prevented the development of natural resistance (Belosevic et al., 1989; Scott, 1991). However, several studies have called into question this fundamental role for IFN-γ in the sequence of events resulting in protection. First, while the administration of anti-IL-4 mAb to susceptible BALB/c mice prevented progressive uncontrolled infection of *L. major* (Sadick et al., 1990) and was paralleled by an up-regulation of IFN-γ production, the concurrent neutralization of this elevated IFN-γ did not abrogate or change the resistance phenotype. Second, mice congenic for known resistance loci (*Lmr1, Lmr2, Lmr3*) derived from either resistant C57BL/6 or susceptible BALB/c mice did not display the expected susceptible/resistant phenotype despite expressing either IL-4 or IFN-γ suggesting that the IFN-γ/IL-4 cytokine profile alone is not a sufficient determinant of disease resistance (Elso et al., 2004a,b; Sakthianandeswaren et al., 2010). Resistant C57BL/6 mice carrying a BALB/c congenic region for *lmr1* displayed increased susceptibility while the reciprocal congenic strain (BALB/c) containing the C57BL/6 allele showed an intermediate phenotype (Elso et al., 2004a). Third, following infection with a *L. major* strain isolated from patient with chronic cutaneous leishmaniasis a non-healing course of disease was observed in C57BL/6 mice despite efficient Th1 polarization (Anderson et al., 2005). Fourth, in our present study we showed that mice deficient for TNF or TNFR1 overexpressed IFN-γ and displayed intact, relatively strong expression of iNOS in the draining LN (Wilhelm et al., 2001), yet, counterintuitively, developed progressive, and ultimately fatal leishmaniasis. While a synergy between IFN-γ and pro-inflammatory mediators (e.g., TNF) based on cooperative signaling through STAT1, IRF-1 and NFκB (Drapier et al., 1988; Ohmori et al., 1997; Saura et al., 1999; Schroder et al., 2004; Farlik et al., 2010) has been described *in vitro* for the production of NO by macrophages (Bogdan et al., 1990; Green et al., 1990; Ding et al., 1998), there appears to be a high level of redundancy *in vivo*. Nevertheless, despite intact induction of iNOS which is associated with parasite clearance (Stenger et al., 1994, 1996; Diefenbach et al., 1998; Blos et al., 2003), *Tnf*^-^*^/^*^-^ mice still failed to control parasite growth and dissemination (Wilhelm et al., 2001).

Taken together, the lack of resistance to *L. major* infection in *Tnf*^-^*^/^*^-^ and *Tnfr1*^-^*^/^*^-^ mice despite an increased production of IFN-γ points to a central role for TNF/TNFR1 signaling in linking innate leishmanicidal effector mechanisms with the adaptive immune response. Since TNF has been shown to modulate the expansion of regulatory T cell networks (Chen et al., 2007), the absence of TNF signaling may modify the local immune response in a way that it becomes refractory to the activity of IFN-γ and iNOS and therefore unable to prevent progressive infection. In addition, the sustained presence of large amounts of systemic IFN-γ throughout the course of leishmaniasis in *Tnf-*negative mice may be the consequence of ongoing infection and parasite dissemination. Instead of promoting protection by activating macrophages the overproduction of IFN-γ could result in immunopathology that contributes to a fatal outcome in response to infection with *L. major*.

## Conflict of Interest Statement

The authors declare that the research was conducted in the absence of any commercial or financial relationships that could be construed as a potential conflict of interest.
